# A Third Ureter Empying into the Urethra. Dripping of Urine. Operation. Recovery

**Published:** 1890-09

**Authors:** 


### A THIRD URETER EMPTYING INTO THE URETHRA. DRIPPING OF URINE. OPERATION. RECOVERY.

From Monatshefte für Prak. Dermats.

On the 29th of March, 1890, Dr. Desiderius Velits presented before the Medical Society of Budapesth the following case from the gynecological clinic of Prof. Tauffer. Is. B., a fourteen-year-old girl, had suffered since birth with dribbling of urine. There was present, on the upper third, left side of the, large, urethra a small opening. A hollow urethral sound directed into this opening upwards and backwards, somewhat to the left, emptied, after a moment, drops of urine. The cystoscopic examination revealed that both ureter openings were normally present in the bladder, and this third canal did not open in the bladder but (shown by moving the catheter) passed over the posterior wall of the bladder, somewhat on the left side—above and to the left. The bladder was opened through epicystomy, and the opening of this third ureter fastened in the bladder, above the sphincter vesicæ, as follows: By means of the inserted hollow sound, the anterior wall of the abnormal ureter was carried behind and above the left vesical ureter opening, and the canal opened with a slit upon the sound, so that the "button" of the sound was now visible in the bladder; and at the same time urine dropped in the bladder through the freshly made opening. Then a portion of this ureter, anterior to the opening, was cut out, and "cut end," cauterized with paquelin. Bladder and abdominal wounds sutured. Healing occurred without reaction, and since (it is about a month after the operation) there has been no dripping of urine. No similar case is recorded in literature.

